# Mechanistic Insights Into Protein Aggregation Inhibition by Green‐Synthesized Silver Nanoparticles: A Study on Human Lysozyme

**DOI:** 10.1049/nbt2/2694374

**Published:** 2026-01-16

**Authors:** Md. Tauqir Alam, Mohd. Ahmar Rauf, Arman Khan, Rizwan Hussain

**Affiliations:** ^1^ Department of Biochemistry, Faculty of Life Sciences, Aligarh Muslim University, Aligarh, 202002, Uttar Pradesh, India, amu.ac.in; ^2^ Department of Biotechnology, Aligarh Muslim University, Aligarh, 202002, Uttar Pradesh, India, amu.ac.in; ^3^ Department of Internal Medicine, Heme/Oncology, University of Michigan, Ann Arbor, 48108, Michigan, USA, umich.edu; ^4^ Department of Industrial Chemistry, Aligarh Muslim University, Aligarh, 202002, Uttar Pradesh, India, amu.ac.in

**Keywords:** Congo red, human lysozyme, protein aggregation, silver nanoparticles, thioflavin T

## Abstract

A characteristic of many neurodegenerative disorders, such as Parkinson’s and Alzheimer’s, is amyloidogenic protein aggregation, for which there are currently no proven cures. Aging, mutation, and physiological stress can cause proteins to deviate from their natural folding patterns, potentially leading to the formation of hazardous protein aggregates. Noble metal nanoparticles (NPs), due to their unique physicochemical properties, have emerged as promising tools in biomedicine, with applications ranging from tissue engineering to drug delivery and diagnostics. Although concerns regarding cytotoxicity exist, small‐sized silver (Ag) NPs (AgNPs) have demonstrated potential in antiviral, anticancer, and antibacterial therapies. This study investigated the development of biocompatible AgNPs using a green synthesis approach and examined their chaperone‐like activity against protein aggregation, emphasizing the role of meticulous in vitro design. Human lysozyme (HLZ) served as a model protein for aggregation inhibition assays. Biogenic AgNPs exhibited a concentration‐dependent effect on HLZ aggregation, demonstrating an optimal inhibitory concentration, followed by a decrease in efficacy at higher concentrations. Furthermore, astrocytes treated with AgNPs displayed reduced protein aggregation, suggesting a chaperone‐like behavior. The initial phase focused on the detailed characterization of AgNPs synthesized using orange juice extract. Subsequently, this study explored the mechanistic understanding of AgNP‐mediated inhibition of protein aggregation under controlled conditions. A battery of biophysical techniques, including circular dichroism (CD), 8‐anilino‐1‐naphthalene‐sulfonic acid (ANS) fluorescence, thioflavin T (ThT) fluorescence, Congo red (CR) assay, and turbidity measurements, was employed to meticulously assess the inhibitory effect on HLZ aggregation in vitro.

## 1. Introduction

Amyotrophic lateral sclerosis (ALS), Parkinson’s disease (PD), Huntington’s disease (HD), Alzheimer’s disease (AD), and prion disorders are neurodegenerative diseases characterized by cellular and molecular processes, including the formation of inclusion bodies and protein aggregation [1, 2]. Amyloids or misfolded proteins with high *β*‐sheet content are frequently observed in these aggregates [3, 4].

Emerging evidence indicates that prefibrillar aggregates, which are precursors to mature fibrils, may be the most harmful species responsible for cellular dysfunction and death in some amyloid diseases [5]. Human lysozyme (HLZ), a well‐understood protein, serves as a useful model for exploring the fundamental principles of protein stability, folding, and structure [6–9]. This bacteriolytic enzyme is found in various tissues and fluids such as the liver, cartilage, plasma, saliva, tears, and milk and specifically cleaves *β*‐1,4 glycosidic linkages within the peptidoglycan cell wall of gram‐positive bacteria [6–9].

Nanotechnology is a rapidly advancing multidisciplinary field that holds significant potential for transformative breakthroughs in medicine. Miniaturization enables the development of smaller, faster, and more cost‐effective mechanical, chemical, and biological components [10, 11]. Beyond size reduction, the unique properties of nanoscale materials include remarkable self‐assembly tendencies driven by forces that differ significantly from those governing macroscopic objects.

Traditional diagnostic and therapeutic approaches for protein aggregation‐related disorders face challenges, such as low sensitivity, specificity, and drug toxicity. These disorders develop over extended periods and are often diagnosed late, limiting the potential for curative treatment. One promising strategy is to prevent protein aggregation by incorporating inhibitory materials into food and beverages [12–14].

Biogenic metal‐based nanoparticles (NPs) have many potential biological uses such as antimicrobial coatings, medical imaging, drug delivery, catalytic water treatment, and environmental sensing [15, 16]. In particular, silver (Ag), gold, and iron oxide NPs have attracted significant attention due to their diverse biomedical potential [17–20].

This study builds upon our prior research on the development of biogenic AgNPs with anti‐aggregation properties. Here, we investigated the effect of green‐synthesized AgNPs on urea‐induced HLZ aggregation under meticulously designed in vitro conditions. The orange juice extract served as a green synthetic agent for AgNPs. Following synthesis, comprehensive characterization using UV‐visible spectroscopy, dynamic light scattering (DLS), zeta potential analysis, and transmission electron microscopy (TEM) was performed to elucidate the nature, size, and shape of the (NPs). Subsequently, the chaperone‐like activity of as‐synthesized AgNPs against HLZ aggregation was evaluated. A series of biophysical assays, including 8‐anilino‐1‐naphthalene‐sulfonic acid (ANS) fluorescence, thioflavin T (ThT) fluorescence, Congo red (CR) absorbance, TEM, and fluorescence microscopy (FM), revealed a concentration‐dependent inhibition of protein aggregation by AgNPs. These findings corroborate our previous observations that aloe vera‐mediated AgNPs prevented *α*‐chymotrypsinogen A aggregation and rose‐mediated AgNPs inhibited HSA aggregation [20, 21].

## 2. Materials and Methods

### 2.1. Materials

HLZ (L1667), ThT, ANS, and CR were purchased from Sigma–Aldrich (USA). The rest of the chemicals used were of analytical grade and were purchased from Sisco Research Laboratories Pvt. Ltd. (SRL), India. Double‐distilled water was used in the preparation of the aliquots.

### 2.2. Methods

#### 2.2.1. Biological Synthesis of AgNPs

The reduction of Ag nitrate (AgNO_3_) was accomplished utilizing orange juice extract to produce green AgNPs. This procedure entailed combining a fixed quantity of AgNO_3_ solution (1 mM) with varying volumes of methanol orange juice ranging from 150 to 500 µL, as described in a previous study [22, 23]. The total volume of the reaction mixture was adjusted to 5 mL through the addition of deionized water. AgNPs were synthesized via continuous stirring at room temperature for a predetermined duration. Subsequently, the mixture underwent centrifugation at 10,000 × *g* for 10 min. This stage is crucial for the elimination of residual plant materials and byproducts. The resulting pellet was further subjected to lyophilization, culminating in the formation of NP.

The as‐synthesized green AgNPs were characterized with high precision utilizing a range of analytical techniques. A preliminary examination was conducted employing spectroscopic analysis. Additional comprehensive studies were performed using TEM (model H‐7500, Hitachi Ltd., Tokyo, Japan) and DLS utilizing a Beckman Coulter Delsa nanoparticle size analyzer (Miami, FL, USA). These methodologies are essential for determining the morphology and dimensions of NP.

#### 2.2.2. Characterization of AgNPs

The biologically synthesized AgNPs were characterized using a range of advanced analytical techniques, as described in the following sections.

##### 2.2.2.1. UV‐Visible Spectroscopy

UV‐vis absorption spectroscopy was employed as the primary tool to assess the bio‐reduction of Ag ions (Ag^+^) to colloidal nanostructures, specifically AgNPs. This assessment was performed using a UV‐visible spectrometer (Hitachi, Tokyo, Japan). This process involved scanning the UV‐visible absorption spectra of the incubation mixtures at various time intervals. This approach is integral to evaluating the kinetics of AgNP formation, providing insight into the rate and efficiency of the biogenic reduction process.

##### 2.2.2.2. Zeta Potential

The stability of the as‐synthesized green AgNPs was determined by analyzing the zeta potential.

##### 2.2.2.3. TEM and DLS Analysis

Two critical methodologies were employed to further characterize the NP. The hydrodynamic particle size of AgNPs was determined utilizing a Beckman Coulter Delsa nanoparticle size analyzer (Miami, FL, USA). This analysis was conducted at ambient temperature to ensure an accurate representation of NP in their native state [24, 25]. Furthermore, the morphology of AgNPs was meticulously examined using a TEM H‐7500 instrument (Hitachi Ltd., Tokyo, Japan). This technique provides high‐resolution images of NP, enabling detailed investigation of their structural properties [26].

#### 2.2.3. Protein Sample Preparation

A stock solution (5 mg/mL) of HLZ was made in sodium phosphate buffer (pH 7.4) and dialyzed overnight. The concentration of protein stock solution was determined using Lowry’s assay [27].

#### 2.2.4. Investigating AgNPs’ Impact on the HLZ Aggregation Pathway In Vitro

##### 2.2.4.1. Turbidity Assay

Turbidimetric analysis was used to conduct a preliminary conformational change analysis in native HLZ in the presence of 6 M urea and increasing concentrations of AgNPs [28].

##### 2.2.4.2. ANS Fluorescence Measurements

ANS is a dye that binds to the exposed hydrophobic segments of polypeptides and is used to investigate protein conformational changes [29].

##### 2.2.4.3. Intrinsic Fluorescence Measurements

A spectrophotometer (RF‐5301, Shimadzu) was used to determine intrinsic fluorescence. The fluorescence spectra were recorded using a quartz cuvette with a path length of 1 cm. The emission spectra were recorded between 300 and 400 nm, and the excitation wavelength was 280 nm [30, 31]. The width of the emission and excitation slits was maintained at 10 nm. The final working concentration of HLZ was 5 μM.

##### 2.2.4.4. ThT Fluorescence Assay

The dye ThT forms a bond with *β*‐amyloids, generating distinctive spectra that have been utilized to study protein aggregation through unfolding for many years. ThT has been considered the benchmark for identifying amyloid fibrils in liquid solutions and tissue secretions for decades [21, 32]. A spectrofluorophotometer (Shimadzu RF‐5301, Japan) was employed to conduct ThT spectral analysis. The researchers used quartz cuvettes with a 1 cm path length. They performed excitation at 440 nm and recorded emission spectra between 450–600 nm. The ThT solution was prepared using double‐distilled water (pH 7.0). The final concentrations used were 20 μM for ThT and 5 μM for HLZ. In this research, the team utilized FM to observe the fluorescence emitted by ThT‐bound HLZ amyloids. ThT is renowned for its capacity to bind to amyloid structures, resulting in a characteristic green fluorescence. This fluorescence occurs due to ThT’s excitation and emission spectrum peaks, which are approximately 450 and 490 nm, respectively. Amyloids from various experimental groups were prepared and placed on a coverslip, which was then mounted on a glass slide. These samples were subsequently examined using FM, specifically employing the fluorescein isothiocyanate (FITC) channel. This method enabled the visualization and analysis of amyloid structures formed under different experimental conditions, highlighting the binding of ThT to these structures.

##### 2.2.4.5. CR Assay

The dye CR, with its chemical composition *C*_32_H_22_N_6_Na_2_O_6_S_2_, binds to the *β*‐amyloids of protein aggregates, resulting in increased absorbance intensity and a spectral shift toward the red end compared to unaltered proteins [33, 34]. A UV‐Vis‐1700 spectrophotometer (Tokyo, Japan) was employed to measure CR absorbance between 400 and 700 nm, utilizing a quartz cuvette with a 1 cm path length. Following a procedure similar to the ThT analysis, the various groups underwent CR‐based imaging, and the prepared slides were examined under FM, focusing on the red fluorescence channel (TRITC).

##### 2.2.4.6. TEM

The morphology of the native and incubated HLZ samples in the presence of increasing AgNP concentrations was examined using TEM.

#### 2.2.5. Statistical Analysis

Each experiment was performed separately in triplicate, and the results are reported as the mean ± SEM. Following one‐way analysis of variance, GraphPad Prism 10.01 (California, USA) was used to analyze the data. Differences were considered statistically significant at P < 0.05 [35].

## 3. Results and Discussion

### 3.1. Green Synthesis and Characterization of AgNPs

Orange peel extract, which is rich in bioactive compounds, such as flavonoids, terpenes, and vitamin C, functions as a reducing and stabilizing agent for AgNP synthesis. These biomolecules reduce Ag^+^ to AgNPs and inhibit their agglomeration. The reduction process was monitored by UV‐Vis spectroscopy (300–700 nm range), and a characteristic shift in solution color from pale yellow to brown/reddish‐brown was observed, indicating nanoparticle formation. The UV‐Vis spectra further confirmed the synthesis of spherical AgNPs by revealing a prominent peak at 425 nm, consistent with the typical surface plasmon resonance (SPR) peak range (410–450 nm) reported for such NP (Figure 1A). TEM analysis confirmed the spherical to oval morphology of the synthesized NP, with a size range of 10–50 nm (Figure 1B). Zeta potential and particle size distribution measurements indicated monodispersity, with an average diameter of 40 nm and an average zeta potential of ‐21 mV (Figure 1C,D). This negative surface charge contributes significantly to the long‐term stability and colloidal nature of AgNPs due to repulsive forces between similarly charged particles.

Figure 1(A) UV‐Visible absorption spectra of AgNPs synthesized using orange peel extract: UV‐Vis spectra of AgNPs generated when AgNO_3_ (1 mM) was incubated with orange peel extract. As the petal extract was added to the incubation mixture, the distinctive surface plasmon resonance (SPR) bands corresponding to AgNPs gradually shifted toward longer wavelengths with associated band intensity amplification. (B) TEM study of as‐synthesized AgNPs reveals their shape and size: A representative TEM image of AgNPs generated from orange peel extract. TEM micrographs demonstrated the coexistence of spherical and oval nanostructures of AgNPs formed during the incubation of orange peel extract in aqueous AgNO_3_. (C) Size study of the AgNPs using DLS: Particle size characterization using dynamic light scattering (DLS) indicates that the as‐synthesized AgNPs have an overall particle radius of approximately 10–50 nm. (D) Zeta potential of synthesized green AgNPs.

**Figure:**
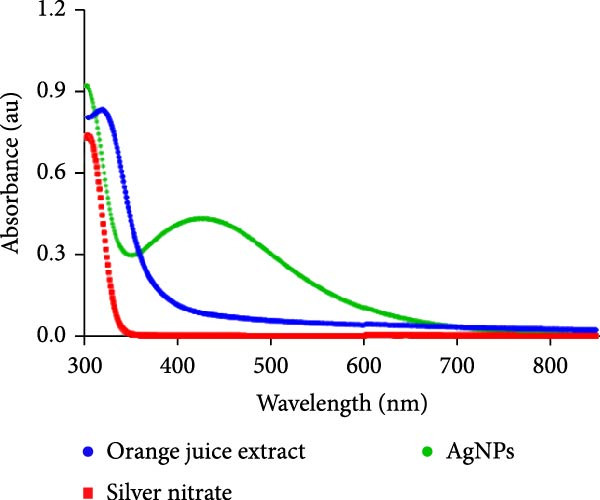
(A)

**Figure:**
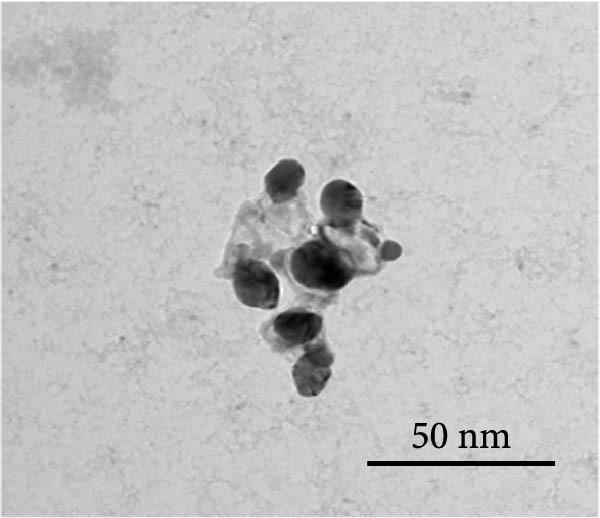
(B)

**Figure:**
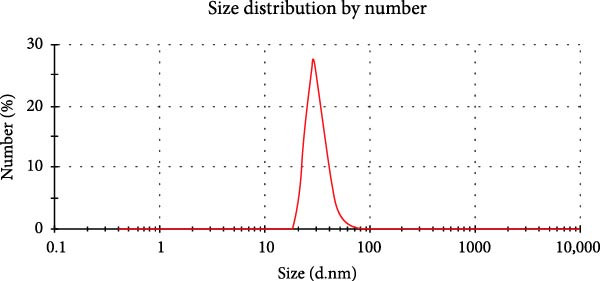
(C)

**Figure:**
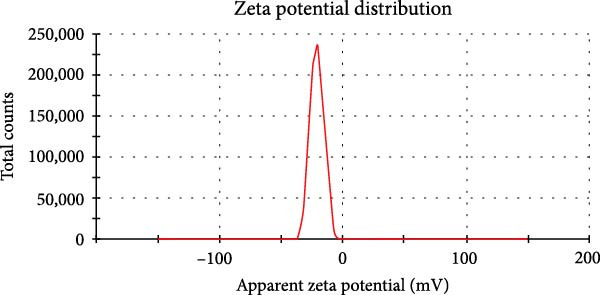
(D)

### 3.2. Investigating Alterations in the Native Structure of HLZ

**Figure 2:**
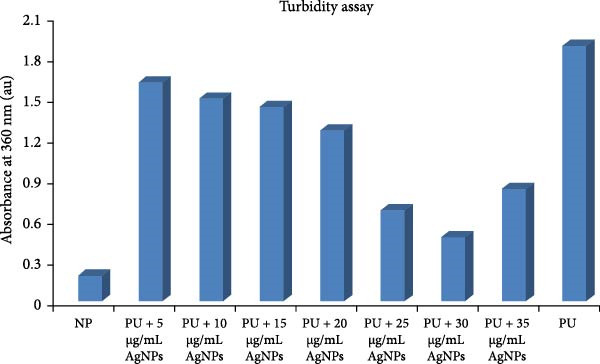
Turbidity assay of incubated samples of native HLZ, HLZ with 6 M urea, and HLA with 6 M urea in the presence of increasing concentrations of AgNPs. All experiments were repeated three times. The final protein concentration was 5 μM, and the turbidity was assayed at 360 nm.

Turbidity assays are frequently utilized in protein aggregation studies as expedient and reliable methods for monitoring aggregate formation. An increase in turbidity is indicative of particle formation, including protein aggregates [36]. Turbidity measurements at 360 nm were initially employed to examine the conformational changes in native HLZ and HLZ incubated with increasing concentrations of AgNPs in the presence of 6 M urea. This investigation aimed to determine the effects of AgNPs on urea‐induced HLZ aggregation at 37°C, the physiological temperature, under controlled in vitro conditions. Turbidity was observed to be lowest for the incubated samples of native HLZ and highest for the HLZ incubated with 6 M urea, due to unfolding‐mediated aggregation induced by urea (Figure 2). A significant decrease in turbidity of the incubated samples of HLZ and urea with green AgNPs was observed in a concentration‐dependent manner, reaching a maximum decrease at 30 μg/mL, above which turbidity increased substantially. These observations suggest that the as‐synthesized green AgNPs possess aggregation inhibition potential, which is most pronounced in the presence of 30 μg/mL AgNPs. This finding was noteworthy for the protein aggregation inhibition potential of the green AgNPs, which was subsequently corroborated using various assays.

**Figure 3:**
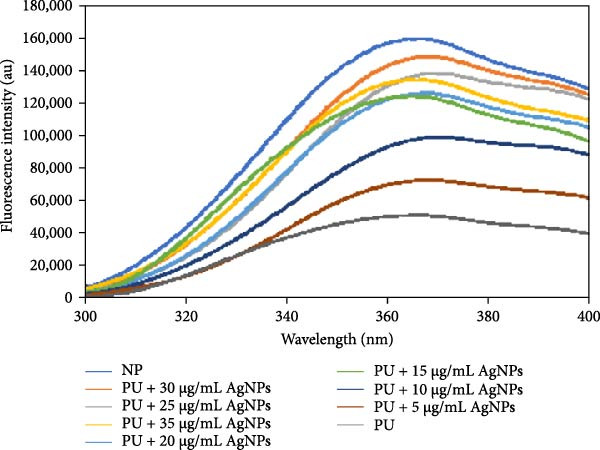
Intrinsic fluorescence emission spectra of native HLZ (curve NP), HLZ in the presence of 6 M urea (curve PU), and the HLZ in the presence of 6 M urea along with increasing concentrations of AgNPs incubated at 37˚C for 24 h in 20 mM sodium phosphate buffer of pH 7.4. The working concentration of HLZ was 5 μM. The excitation wavelength was 280 nm, and the emission wavelength ranged from 300 to 400 nm. The excitation and emission slit widths were chosen at 10 nm.

Intrinsic fluorescence spectroscopy is a valuable tool for studying protein conformational change analysis [37]. This technique is based on the intrinsic fluorescence capabilities of aromatic amino acid residues, which emit fluorescence when excited by ultraviolet light. The intrinsic fluorescence of HLZ is attributed to the presence of five tryptophan, two phenylalanine, and six tyrosine residues [38]. The intrinsic fluorescence intensity of native HLZ was observed to reach its maximum peak at 365 nm, whereas the intrinsic fluorescence intensity of the HLZ sample incubated with 6 M urea exhibited a sharp decline (Figure 3 curves NP and PU, respectively). The intrinsic fluorescence intensities for the HLZ samples incubated with 6 M urea in the presence of AgNPs were found to be enhanced, approaching the native spectra in a concentration‐dependent manner up to 30 μg/mL, after which a significant decline was observed. The decrease in the intrinsic fluorescence quenching results indicated that the green AgNPs demonstrate maximum protein aggregation inhibition potential at approximately 30 μg/mL, above which the effect of protein aggregation inhibition is reduced. These results were consistent with those of the turbidimetric assay and were further corroborated by ANS fluorescence analysis. In our previous research, we demonstrated that when HSA samples were incubated at a temperature of 40°C with 5 M urea in vitro, AgNPs (size 10–50 nm) maximally inhibited protein aggregation at 30 μg/mL. In the present study, when HLZ was incubated with 6 M urea at a physiological temperature (37°C), we observed that the AgNPs inhibited protein aggregation maximally at 30 μg/mL.

**Figure 4:**
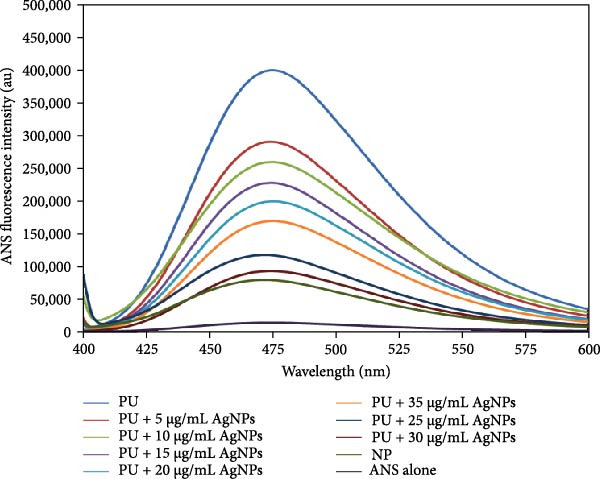
ANS fluorescence spectra of incubated samples of native HLZ, HLZ with 6 M urea, and HLZ with 6 M urea in the presence of increasing concentrations of AgNPs. The excitation and emission wavelengths were 380 nm and the range 400–600 nm. The excitation and emission slit widths were both set to 10 nm.

Extrinsic fluorescence utilizing ANS is a robust method for examining conformational changes in proteins, including modifications to hydrophobicity and exposure to hydrophobic areas [39, 40]. This technique is particularly effective for monitoring the unfolding or misfolding of proteins under various experimental conditions. ANS binds to the hydrophobic regions of polypeptides. ANS fluorescence spectral measurements provide information regarding the alterations in the native structure of proteins [41, 42]. The incubated sample of native HLZ exhibited minimal ANS fluorescence intensity with a peak at approximately 470 nm, indicating that the majority of the hydrophobic residues were internalized, whereas the incubated HLZ sample in the presence of 6 M urea demonstrated approximately five fold increased ANS fluorescence intensity with a peak at approximately 477 nm (Figure 4, curves NP and PU) compared to that of native HLZ, suggesting that the native HLZ had unfolded, resulting in increased ANS fluorescence intensity. The spectra of incubated HLZ and urea in the presence of green AgNPs revealed a lower ANS fluorescence intensity, which approached that of the native. The incubated samples of HLZ with 6 M urea in the presence of increasing concentrations of AgNPs exhibited quenched ANS fluorescence intensity approaching the native HLZ, and the maximum ANS fluorescence quenching was observed for the sample with 30 μg/mL AgNPs, indicating maximum inhibition of protein aggregation at this concentration in an in vitro environment.

### 3.3. Validating the HLZ Aggregation and Aggregation Inhibition

ThT assay is a widely utilized method for detecting and quantifying protein aggregation, particularly amyloid fibril formation [43, 44]. This assay relies on the capacity of ThT, a benzothiazole dye, to specifically bind to *β*‐sheet‐rich structures, which are characteristic of amyloid fibrils and aggregates. When ThT binds to these structures, it undergoes fluorescence enhancement, enabling the detection of protein aggregates. The ThT fluorescence intensity of the native HLZ was observed to be the lowest, whereas the HLZ sample incubated with 6 M urea exhibited a more than fivefold increase in ThT fluorescence intensity (Figure 5A, curves NP, and PU). The elevated ThT fluorescence in HLZ treated with 6 M urea could be attributed to the presence of cross‐*β*‐sheets, a key characteristic of protein aggregation [14, 45]. It is noteworthy that incubating HLZ samples with 6 M urea in the presence of increasing concentrations of green AgNPs resulted in substantial quenching of ThT fluorescence intensity (Figure 5B), approaching that of native HLZ in the presence of 30 μg/mL AgNPs. These findings suggest that the green AgNPs demonstrate protein aggregation inhibition potential at a concentration of 30 μg/mL.

Figure 5(A) ThT fluorescence spectra of native HLZ, HLZ with 6 M urea, and HLZ with 6 M urea in the presence of increasing concentrations of AgNPs. The final concentration of HLZ used was 5 μM while that of ThT was 20 μM. 5 (B) Micrographs captured through fluorescence microscopy provided a visual comparison of the HLZ amyloids under different conditions: (B1) The HLZs in native form without amyloids formation, (B2) The samples contained HLZ amyloids without any AgNPs, whereas the other set included the HLZ amyloids incubated with AgNPs (B3). This comparative analysis was experimental in determining the influence of AgNPs on the formation and structure of HLZ amyloids, with green‐colored fluorescence serving as a clear marker of the presence and extent of amyloid formation.

**Figure:**
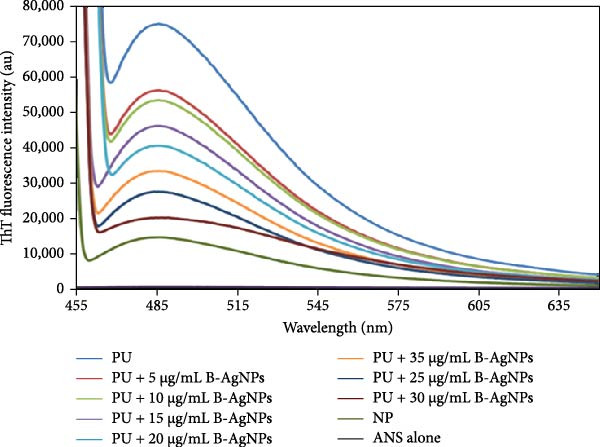
(A)

**Figure:**
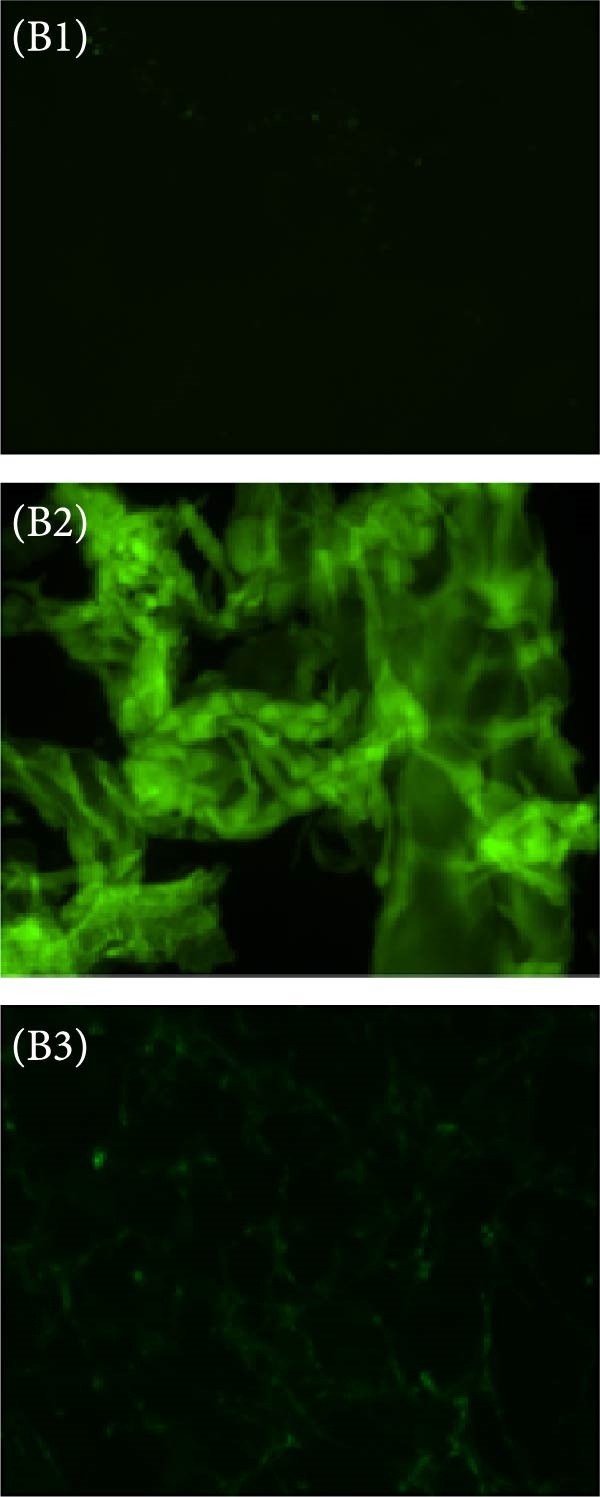
(B)

The CR absorbance assay is another widely utilized method for investigating protein aggregation, specifically the formation of amyloid fibrils. Analogous to the ThT assay, this technique relies on the binding of the CR dye to *β*‐sheet‐rich structures in protein aggregates [46]. The interaction between CR and *β*‐sheet‐rich protein aggregates results in a characteristic red shift in the absorbance spectrum, which can be monitored to quantify and analyze protein aggregation [47–49]. CR absorption spectral analysis confirmed the development of cross‐*β*‐sheet‐rich HLZ aggregates in the presence of 6 M urea and the aggregation inhibition potential of the green AgNPs. In this investigation, it was observed that the native HLZ exhibited minimal absorbance spectra, with an absorbance peak at approximately 498 nm that was amplified threefold with a red shift to approximately 505 nm. This increased CR absorbance with a red shift indicates HLZ aggregation. Furthermore, the samples of HLZ incubated with 6 M urea in the presence of increasing concentrations of green AgNPs approached native HLZ and demonstrated a significant decrease in CR absorbance (Figure 6A). Additionally, the decrease in CR absorbance was most pronounced for the HLZ sample incubated with 30 μg/mL AgNPs, suggesting maximum protein aggregation inhibition potential at this concentration compared to the untreated and native protein (Figure 6B [A–C]). These findings provide substantial evidence for the protein aggregation inhibition potential of green AgNPs, which may be considered as potential therapeutic agents following further evaluation in animal models.

Figure 6(A) CR absorption spectra of native HLZ, HLZ with 6 M urea, and the HLZ with 6 M urea in the presence of increasing concentrations of AgNPs. The working concentration of HLZ was 0.8 μM and the path length was 1 cm. (B) Fluorescence microscopy micrographs showing the as‐synthesized HLZ amyloids under two different conditions: (B1) Native HLZ without amyloid form, (B2) the HLZ amyloids in the absence of AgNPs, and (B3) the HLZ amyloids in the presence of AgNPs. These micrographs provide visual evidence of how the inclusion or exclusion of AgNPs affects the conformation and structure of the HLZ amyloids.

**Figure:**
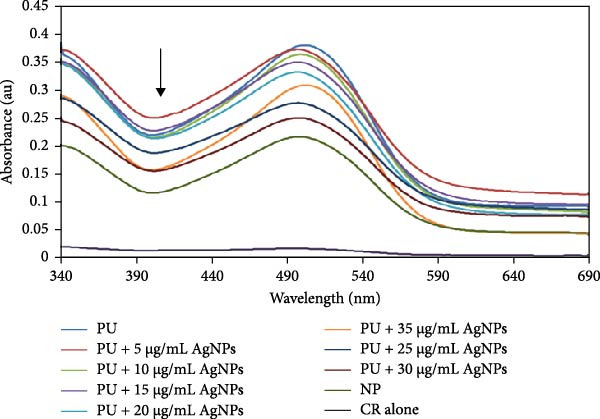
(A)

**Figure:**
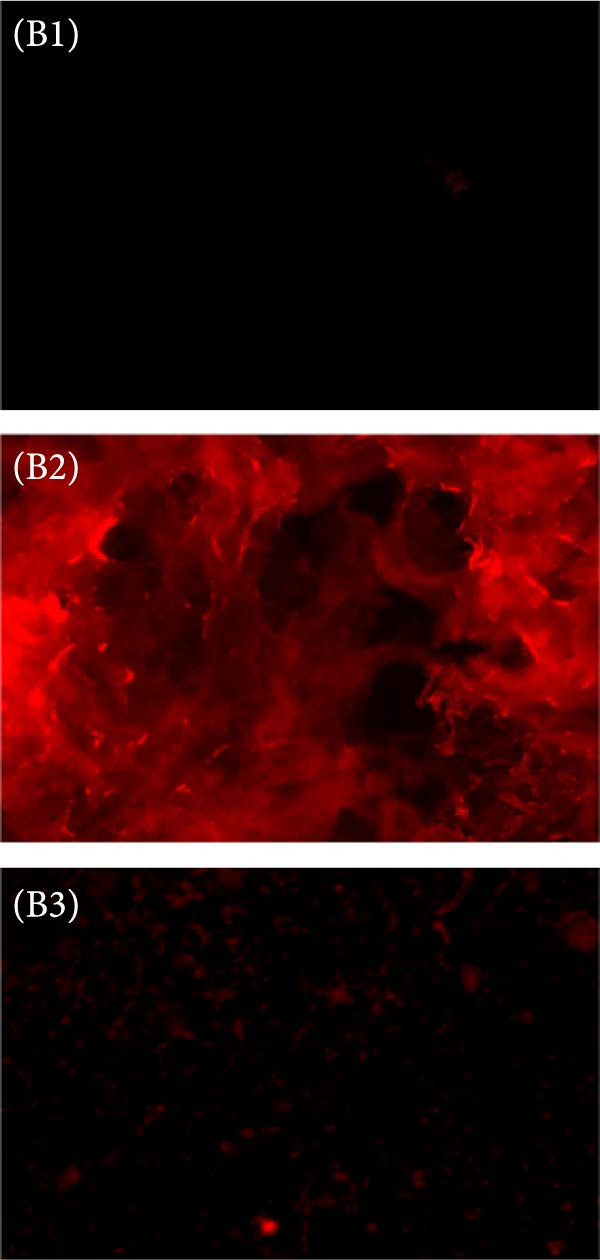
(B)

### 3.4. Morphological Analysis of Aggregates

TEM (Figure 7) analysis provided direct visualization of AgNP‐mediated inhibition of protein aggregation. Native HLZ samples exhibited no signs of aggregation (Figure 7A). In contrast, significant aggregation was evident in the 6 M urea‐treated sample (Figure 7B). Figure 7C reveals a substantial reduction in protein aggregates upon co‐incubation of HLZ with 30 µg/mL AgNPs, demonstrating the NP’ capacity to suppress protein aggregation. This inhibitory effect of AgNPs on protein aggregation exhibited concentration dependence. While increasing AgNP concentration up to 30 µg/mL enhanced aggregation suppression, concentrations exceeding this threshold did not yield further improvement (data not shown). This observation suggests an optimal concentration range for AgNP‐mediated aggregation inhibition activity. This study elucidates two key findings: the potential of AgNPs to mitigate protein aggregation and the critical importance of optimizing AgNPs concentration for maximal therapeutic efficacy.

Figure 7Transmission electron microscopy of incubated samples of native HLZ, HLZ with 6 M urea, and HLZ with 6 M urea in the presence of 30 μg/mL AgNPs (A, B, and C, respectively).

**Figure:**
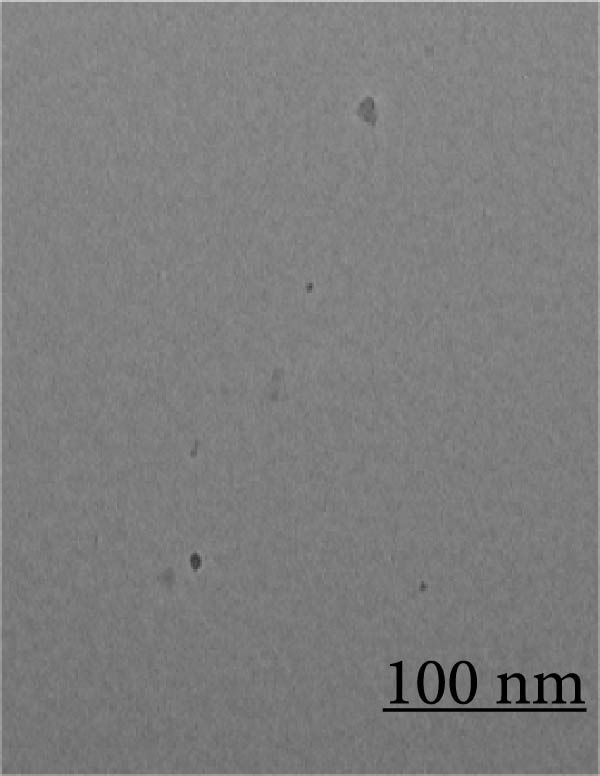
(A)

**Figure:**
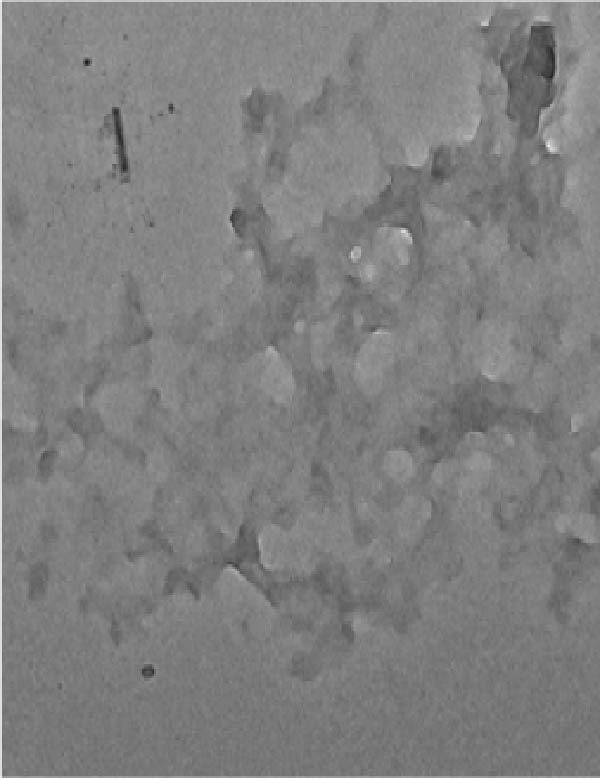
(B)

**Figure:**
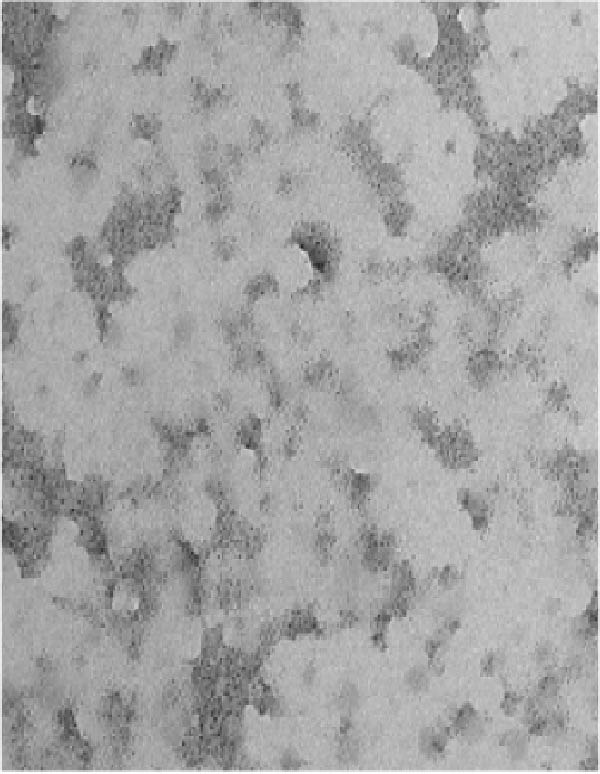
(C)

### 3.5. The Protective Effect of AgNPs Against Cytotoxicity Induced by HLZ‐Amyloidal Aggregates in Astrocytes

**Figure 8:**
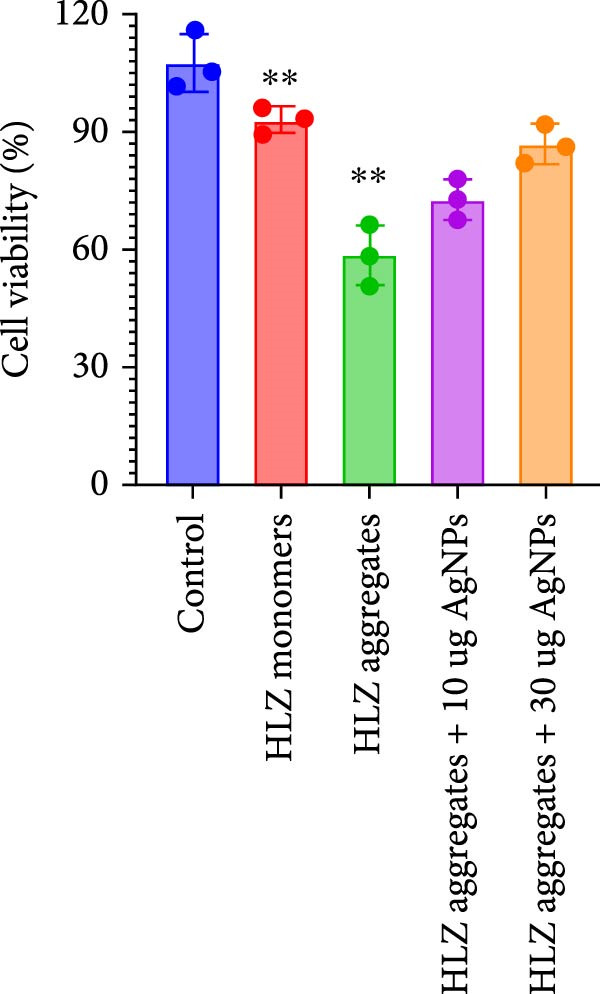
The MTT (3‐(4,5‐dimethylthiazol‐2‐yl)‐2,5‐diphenyltetrazolium bromide) assay for cell viability was employed in this study to evaluate the effectiveness of as‐synthesized AgNPs in inhibiting the formation of hazardous HLZ aggregates. Cells were exposed to HLZ fibrils both with and without the addition of AgNPs. This assay provided a quantitative measure of the protective effect of AgNPs against cytotoxicity associated with HLZ aggregate formation.

The cytotoxic impact of HLZ amyloid aggregates was assessed using a Cell Titer Glo assay over 48 h. Our results revealed a significant reduction in cell viability to approximately 64%, indicating the detrimental effect of these aggregates on cells. Notably, the co‐incubation of HLZ amyloidal aggregates with AgNPs at concentrations of 10 µg/mL and 30 µg/mL resulted in a dose‐dependent increase in cell viability to 68% and 78%, respectively (Figure 8). This enhanced viability can be attributed to the anti‐amyloidogenic properties of AgNPs. We posit that AgNPs disrupt amyloid aggregation, a key factor in the observed cellular toxicity. The formation of insoluble amyloid aggregates is a primary driver of cytotoxicity in living cells. Our data suggest that AgNPs may impede amyloid formation by potentially extending the lag phase, particularly by targeting early aggregation intermediates. AgNPs might achieve this protective effect by sequestering these harmful clusters, thereby depleting the pool of available precursors for amyloid assembly. This mechanism aligns with the observed mitigation of cytotoxicity by AgNPs in astrocytes exposed to HLZ amyloid aggregates.

The application of biogenic AgNPs within astrocyte cultures offers valuable insight into their potential therapeutic use. Our findings indicate that AgNPs promote a significant reduction in astrocyte toxicity, likely due to their capacity to mitigate the formation of harmful protein aggregates. This aligns with our previous investigations, which demonstrated the cellular uptake of AgNPs within astrocytes. We hypothesize that upon internalization, AgNPs exhibit chaperone‐like behavior, actively preventing protein misfolding—a key pathological process in numerous neurodegenerative diseases. Data from cell viability assays strongly support the anti‐aggregation properties of AgNPs, demonstrating a dose‐dependent ability to prevent harmful protein cluster formation. Consequently, varying AgNP concentrations directly influence astrocyte survival under conditions of protein aggregation stress. Our study underscores the therapeutic potential of AgNPs in minimizing the deleterious effects of protein aggregation within brain cells. The application of biologically produced AgNPs in astrocyte cultures has provided valuable information regarding their potential therapeutic uses. The ability of AgNPs to mitigate dangerous clusters results in a significant reduction in toxicity in astrocytes. This detoxifying action was confirmed in our previous investigations, which demonstrated that when astrocytes were exposed to AgNPs, the NP were internalized by cells. It has been postulated that when internalized by astrocytes, AgNPs may function as chaperones, actively preventing the misfolding of proteins. This role is critical because protein misfolding can lead to a range of neurodegenerative diseases. The data obtained from the cell titer experiments strongly support the hypothesis that AgNPs possess anti‐aggregation properties. These NP can inhibit the formation of harmful clusters, and this ability increases with the dosage. As a result, applying varying concentrations of AgNPs has a direct effect on and influences the survival rates of astrocytes exposed to these conditions. Our findings emphasize the therapeutic potential of AgNPs in attenuating the negative consequences of protein aggregation in brain cells.

## 4. Conclusions

This study investigated the concentration‐dependent influence of green‐synthesized AgNPs on potential conformational changes in HLZ. The extensive application of AgNPs in pharmaceuticals and food necessitates a comprehensive understanding of their interactions with biomolecules. Our findings demonstrate that the synthesized AgNPs effectively inhibit HLZ aggregation up to a concentration of 30 µg/mL, with a decrease in efficacy at higher concentrations. This underscores the importance of considering concentration‐dependent effects when utilizing green NP. While this work represents a preliminary investigation of AgNP‐mediated HLZ aggregation suppression under controlled in vitro conditions (employing urea), it acknowledges the limitations of replicating in vivo complexities. The natural environment presents numerous proteins and other factors that could influence HLZ interactions. Despite these limitations, this study establishes a foundation for future research aimed at elucidating the role of AgNPs as potential inhibitors of protein aggregation, amyloid fibril formation, and unraveling the mechanisms underlying their interaction with amyloidogenic proteins.

**Nomenclature**
HLZ: Human lysozyme
AgNPs: Silver nanoparticles
ThT: Thioflavin T
CR: Congo red
CD: Circular dichroism
TEM: Transmission electron microscopy
FM: Fluorescence microscopy.

## Conflicts of Interest

The authors declare no conflicts of interest.

## Author Contributions

Md. Tauqir Alam and Mohd. Ahmar Rauf contributed equally to this work.

## Funding

The authors did not receive any specific grant or funding support for this work from public, commercial, or not‐for‐profit funding agencies.

## Data Availability

The data supporting the findings of this study are available from the corresponding author upon reasonable request.
